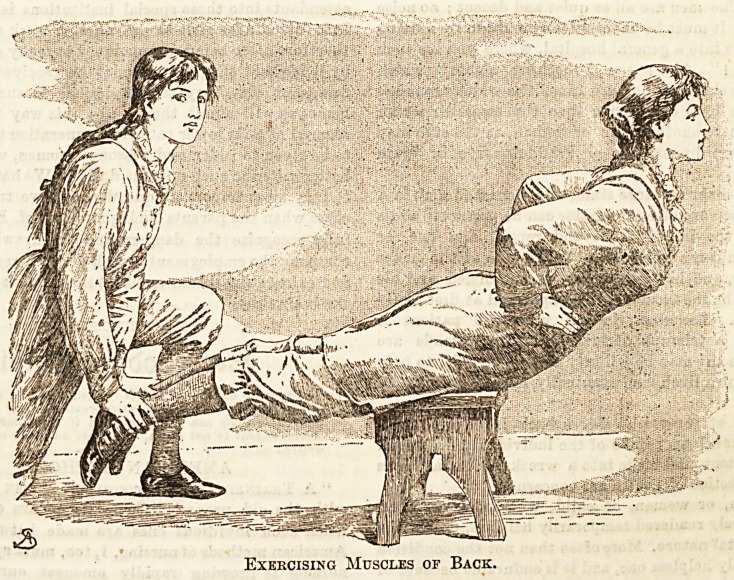# The Hospital Nursing Supplement

**Published:** 1893-02-04

**Authors:** 


### "The Hospital" Nursing Mirror.

Being the Extra Nursing Supplement of "The Hospital" Newspaper.

[Contributions for this Supplement should be addressed to the Editor, The Hospital, 140, Strand, London, W.C., and should have the word "Nursing" plainly written in left-hand top corner of the envelope.]

### News from the Nursing World.

SECOND EDITION, Thursday Morning.

### THE INTERNATIONAL NURSING CONGRESS, CHICAGO.

We received the following communication from Miss Isabel A. Hampton, Chairman of the Nursing Section of the Congress, after we had gone to press. It is so important, however, that we have determined to give it in a Second Edition. May we venture to hope, and to urge once more, that all who have a claim to be regarded as amongst the leaders of nursing in this country will combine to make the first Nursing Congress a great success. If wise counsels prevail, the Chicago Congress, as a neutral centre, where all can meet on equal terms, may result in reconciling conflicting interests and views to the no small advantage of sound nursing all the world over.

Dr. Hurd has shown me a telegram from Dr. Billings in which you ask if I had appointed Mrs. Bedford Fenwick to organise the British section of the International Congress of Nurses. The reply was that I had not. Permit me also to explain that as the International Nursing Congress was first planned, Mrs. Fenwick was appointed by the Chicago Committee as chairman of the British section. After it became part of the section on hospitals, &c., it was not thought necessary to have the appointment continue. Consequently, I am arranging in England and all Great Britain for papers and discussions, and am writing to the people myself. In doing so I am not giving Mrs. Bedford Fenwick, the Nurses' Association, nor the Nurses' Pension Fund members any special consideration, but am asking only for the co operation of all British nurses themselves. Any unpleasant feelings or conditions that may exist among themselves will not, I trust, be brought in any way into the Congress, which should be of interest and benefit to all nurses. I write you this as you are the editor of a nursing journal, and I should be sorry to have any statements appear through misunderstanding.

### AN ACCEPTABLE GIFT.

Her Majesty the Queen has been graciously pleased to send a present of cast linen for the use of the patients of the Grosvenor Hospital for Women and Children, Vincent Square.

### THE HOSPITAL FOR SICK CHILDREN.

Their Royal Highnesses the Prince and Princess of Wales have graciously consented to open the new wards at the Hospital for Sick Children in Great Ormond Street. The ceremony will take place in June next.

### A PLEASANT PRESENTATION.

Miss Walker, better known as Sister Dorothy, left the London Hospital last week to take up other work. Sister Dorothy has filled the arduous post of Home Sister for four and a-half years, and she received, on her retirement, many kind proofs of the affection which she has gained from her fellow-workers. Perhaps one of the most gratifying took the form of an address, which accompanied a handsome writing-case and other useful gifts from the private nursing staff. The dainty card bore the following words in gold letters: "Please accept these small tokens of our appreciation of the kindly welcome which the members of the private staff, past and present, have always received from you on each return to the Nursing Home," &c. This is the kind of satisfactory offering of which mention was lately made in the The Hospital under the heading of "Presents and Presentations." The sentence ran thus: "The motive of a presentation to a person on the eve of departure admits of no criticism."

### GIRL-NURSES.

"An Experienced Nurse" contributed a letter last week treating with much good sense on a very important question. In the majority of training schools the girl-nurse has become an impossibility, for even the weekly guinea does not gain her admittance to St. Thomas's, King's College Hospital, nor the London. All large hospitals have for some time past ruled the age for admission of probationers to be twenty-five years. Incurable Homes, Asylums, and Fever Hospitals, as well as some few Infirmaries, unfortunately still encourage the girl-nurse. Our experienced correspondent says truly that parents know little of what lies before their young daughters when they exchange their home life for the duties of these institutions.

### WHOSE FAULT IS IT?

Every week new societies are formed for supplying trained nurses to the sick poor, and most of the associations are excellently managed. They aim at good work, and generally attain to it. Yet now and then transactions take place which strike outsiders as peculiar ones. For instance, although the district nurse may be fully occupied with her own lawful work, when the wife of a rich neighbour falls sick, she at once claims the services of the familiar nurse. In fact, she refuses to entertain for a moment the idea of any other attendant. The doctor sees how undesirable it is to interfere with the parish nurse, for, in a general way, he knows she has plenty to do; but even he does not realise how arduous and unceasing are her duties. However, a country practitioner cannot often afford to displease a rich patient, so he gives in—says he will "ask nurse to come in for the present." The result can be guessed at. The rich woman secures free nursing, and never dreams of resigning her patient attendant, and the latter sees her "extra case" safely through her illness, doing her own regular work at odd times, and by and by breaks down. Perhaps she has to pass some weeks of invalidism at her own expense. All this waste of strength could be avoided if the patient, who has no right to a public nurse, were forced from the first to engage a private one. There should be a rule in all district nursing associations, and in cottage hospitals too, that nurses engaged for the service of the poor may not be annexed under any pretext whatever by other persons. The doctors acquiesce, under compulsion, in an arrangement to which their consciences can never give cordial consent.

### WISE DONCASTER.

At Doncaster the other day the question of appointing a district nurse was laid before the governors of the infirmary and dispensary. The step seems a particularly wise one, for the nurse they decided to engage will work under the direction of the medical staff amongst the dispensary patients. She will attend them in their own homes, and do such dressings as may be required, and otherwise see that the doctors' orders are carried out. It is impossible to measure the value of such services as these, the benefits to the patients, and the assistance to the medical men, being alike unlimited. There is but little satisfaction in giving orders which are not likely to be carried out, and yet that is just what the doctors in the out-patient departments have to do daily. It would be economical, as well as merciful, if all hospitals saw their way to maintaining a nurse for the out-door department. Many a patient has to be admitted to the ward because he cannot get the needful attention at home, yet there will always be cases which would do equally well in their own dwellings, if a skilled person could undertake the "outward applications," which can be rendered positively harmful in ignorant hands. Certainly competent district nurses possess a very wide field of usefulness, and their valuable services are increasingly in demand.

### TRAINED NURSES FOR WIRKSWORTH.

At Wirksworth a public meeting was recently held to consider the proposal of the Local Government Board for engaging a sick nurse for the poor. If the guardians exercised their power of establishing one, she would be available only for persons receiving parish relief, and those not officially "paupers" could not be nursed by her, whatever the urgency of the cases. Dr. Harvey suggested that the guardians should have a nurse for the paupers, whilst the Board might supply one for the other necessitous poor. The Chairman suggested a plan for obtaining a nurse from some home when necessity arose, as in epidemics. Unhappily sickness generally is in itself epidemical amongst the poor at all seasons, and experience shows that district nurses are generally employed to a certain extent during every month of the year. An element of comedy was introduced into an otherwise serious assemblage by a certain Mr. Gamble, who apparently holds the opinion that nursing is not a matter of training—or is it that Wirksworth is singularly blessed in "born-nurses"? At any rate he is reported to have said he "objected to the introduction of trained nurses. He considered they had plenty of women in the town competent to act as nurses, and who would be glad to do so for payment without sending for any fine lady with a long veil down her back. If money was to be spent let it by all means be spent in the town." This sounds well, and it is to be hoped that other home-manufactures are consistently supported in Wirksworth. It is, however, lucky for the sick poor that the majority of rate payers voted in favour of trained nursing, and appointed a committee composed of members of the board as well as guardians to carry out the plan.

### ENGLISH NURSES AT SAN REMO.

The English nurses at Miss Bryant's Institute at San Remo are all very busy just now, and additional workers have been engaged. Doctors appreciate the advantages of having their orders faithfully carried out, and English invalids are thankful to find their own trained countrywomen ready to nurse them in a foreign land. A pleasant flat with a lovely view of sea and hills forms the headquarters of this useful little colony of English nurses.

### EUROPEANS IN INDIA.

The very word India suggests wealth and luxury. Perhaps this is the reason few people seem to grasp the fact that many of the Europeans residing in that hugh empire are by no means rolling in wealth. The question of securing trained nursing at a reasonable cost for these people with moderate incomes has called forth not only discussions, but certain plans. The *Times of India* urges the European community in each Indian Presidency to respond and co-operate in a scheme for securing skilled attendance for the sick in up-country districts. There is no prospect of fortunes being made by nurses who respond to this call, but they ought to be able to reckon upon a fair livelihood. Certainly the class of probable employers must be able to pay a moderate fee punctually. Still, it should not be forgotten that modest remuneration is not much compensation to offer a nurse in exchange for the exile which life in an up-country station must mean for her. If good nurses are willing to give up their familiar surroundings and their home circles, and to devote their talents to the service of Europeans in India, they must be well treated. Probably they will not be able to stand many years' work in that country, and if they return home invalided they, in their turn, will need nursing. To join the R.N.P.F., which has a "sick pay" department, ought to be a preliminary step taken by every nurse ere accepting a foreign nursing appointment. Civilians are apt to speak jealously of the better fortune of the military as regards the luxury of trained nursing, but "Tommy Atkins" certainly seems to consider that more attention might advantageously be paid to his needs in remote and unattractive stations.

### A NEW NURSES' HOME.

Amongst the many excellent arrangements at the Prince Alfred Hospital, Sydney, the new Nursing Home contains a kitchen, where the probationers will receive instruction in invalid cookery. The Home is most complete, every comfort and convenience having been wisely provided for.

### DISABLED NURSE.

Each subscription has been accompanied by kind words and good wishes for our suffering Sister, and we venture to hope that the latter will be realised, and that she may eventually be restored to better health. We beg to acknowledge the following sums: Nurse M. Thomas, 3s. 6d.; No. 1,238, 4s.; Sister Moira, 5s.; Nurse May, 2s. 6d.; Nurse Florence, 1s.; Nurse Janet, 1s.; Nurse Elena, 1s. 6d.; A Sympathiser, 5s.

### "THE HOSPITAL" ENDOWED BED.

We beg to remind our readers that many of the annual subscriptions to the Nurses' Bed are still unpaid. We shall be glad to receive them with as little delay as possible. Miss R. Pritchard has sent £1; Miss Heanley, 4s.; Miss L. E. Smith, £1 1s.

### The Development of Children by Gymnastics.

### VII.—LING'S SYSTEM.

Another branch of Ling's system introduces the aid of a second person. There are many movements which it is impossible to make without outward support; our illustration shows one of these exercises in progress. One pupil rests across a stool, chair, or desk so that the upper part of the body may be free to move from the hip joints; another pupil then places her hands firmly on the ankles of the girl exercising, and uses just enough resistance to prevent her overbalancing. The first pupil then places her hands on her hips and raises the body from the waist, curving the back as much as is conveniently possible, then bending down till the body is again in a straight line with the legs. This is repeated three or four times, and serves as a capital exercise for all the muscles of the back.

An exercise similar to this is introduced into the medical section of the Ling system, but the pressure is here increased by an attendant, who forces the shoulders back further than is possible by the unaided efforts of the patient, and when bending downwards the upper part of the body is allowed to hang at right angles to the legs. These medical gymnastics, however, necessitate the attendance of so many helpers that they are hardly practicable in ordinary cases. Somewhat the same results are aimed at by the Zander system, where the patient is placed on machines which, in some instances, move so as to exercise certain limbs, and in others take the place of the assistants in the Ling system and "hold" the patient while he makes the movements. These courses, however, come more under the head of medical treatment than that of the mere training of children, and though they have proved most beneficial in correcting disease, yet we advocate for the young a course which brings into play their own motive powers, in the using of which they become self-reliant and precise in action.

Exercising Muscles of Back.

As to the advisability of choosing the Ling or German system for his or her particular children it is for the parent or guardian to decide, but that a choice of some such course of physical training is necessary I hope will by this time be evident to all. The gymnasium generally meets with a warmer welcome from the British boy than does the more novel Swedish system, perhaps because youth is ever eager to accomplish the same feats as former athletic heroes, perhaps for the pleasure of mastering the exercises themselves.

It is urged by German gymnasts that the culture of the body by their system serves to make it an able and obedient servant of the will, and that this is accomplished by a rotation of exercises the performance of which evades the tediousness of exercising each group of muscles separately, they maintain also that their exercises train the various muscular groups to work in harmony as in every day life, and not merely to bring each part to its highest possible development.

On the other hand, the Ling system undoubtedly presents itself in a more favourable light to the heads of schools, and especially those of the usual moderate size, since the important item of apparatus is practically nil with the free movements, and the ordinary school furniture—tables, chairs, forms, desks—supplies all that is wanted for the advanced Ling exercises. Besides its convenience, the Ling system claims to be the more gradual and, therefore, more lasting results than the other schools. No exercise is carried beyond rational limits. The main rock on which it is founded is that all exercise must begin and terminate gradually, "the greatest muscular effort must be in the middle," and the exercises chosen must be in a regular course, one group of muscles being rested whilst another is in play.

On the advocates of these various systems it may be urged that they defeat not their own ends by controversies as to the best "school." The great international movement in favour of physical education is beginning to show good practical results. It is to be hoped its promoters will not check its progress by the petty bickerings and narrowminded jealousies which so often bring to naught the most noble efforts in world-history. The ultimate aim, a sound harmonious body obeying promptly the dictates of a well trained will, and striving to fulfil its part of the "complete being," the aim to which all education tends, and one set forth by both schools, is one so great and worthy that the efforts of the one, marred though they may be by occasional imperfections, should command the sympathies and forbearance of the other—true greatness of character forgets all rivalry in the joy of feeling that the desired goal is reached even if it be by other roads than its own; if we succeed in restoring the human frame to that state of perfection it was designed to attain we surely care not by whose *method* it is effected—but for that end we must strive, and it is the solemn duty laid on those who have charge of the young that they do all in their power to cherish, tend, and develop the body, that it may aid rather than hinder the development of the mind, that it may be a worthy temple of the intellect. Let them supply the strong, muscular, well-developed frame, and it will be found that health, grace, agility, nay, even largeness of character and an open, honest heart will come with it, and this once formed it will be but an easy and pleasant task to keep up the "tone." If we can accomplish this it will be with more than gratitude that our children will look back on our memory, and we shall have done the duty set before us and conferred a lasting benefit on generations to come.

### Helpless Patients.

The word "helpless" is seldom appreciated in its full meaning by any save trained nurses or other attendants on paralysed and such-like cases. We say to a stranger, in answer to a question, "Yes, this poor man is absolutely helpless, he cannot do anything for himself;" and we hear the comment, "Dear me, how sad! does he really never move from his bed?" and the matter drops. If the visitor has no imagination she goes away quite satisfied that a ward full of such patients, some of them speechless, is really quite an unobjectionable field for the labours of a young girl in whom she is interested. "The men are all so quiet and decent; no noise or loud joking; it must be much better to begin by nursing them than to go into a general hospital, where you see such shocking sights!" Alas! the "shocking sights" which ultra-refinement shrinks from, are casualties which are much less sad in the eyes of a nurse than the stretcher which deposits in her care another man or woman, as the case may be, stricken down by sudden or gradual nerve or brain disease.

A little blood makes a large stain, and a crushed limb is a pitiful sight, but very often the latter can be preserved to its owner. When the injury is beyond repair, and one, or perhaps two, limbs are sacrificed, even then the victim makes a good recovery, and his intelligence is undimmed and his spirit unbroken by the accident which appeared at first likely to imperil both. Therefore, in the ward the patient is looked upon as a triumph of surgery, many friends are interested, and some may be willing, as well as able, to help him to earn his own livelihood eventually, despite his maimed condition.

The sensitive stranger who draws back from the blood-stained garments seldom thinks of the individual thus struck down in a moment and made into a wreck for life, as far as vigour and perfection of health are concerned.

But the man, or woman, or child, who meets with an accident, is merely rendered temporarily helpless, unless the injury is of a fatal nature. More often than not the condition is only a partially helpless one, and it is endurable because of its limited duration.

But when we say "helpless" of infirmary patients, of those in "Homes for Incurables," or chronic cases in hospitals for nervous diseases, &c., the word has a terrible significance in the ears of experienced nurses. They know the poor creatures have often acquired most grievous bed-sores before their friends have succeeded in passing them into skilled keeping, and they are aware, too, that it will be a slow and tedious business to heal these, and that in many cases such cures will never be made. The patient will lie like a log wherever he is placed, and for months to come he will not have power to to rid himself of one straying crumb or of one intrusive fly! And the fly knows it, too, or at any rate he acts as if he did, for his most irritating onsets are made on those least able to parry his attacks. Helplessness in a child is natural, and therefore attractive; in a man or a woman it is the most pitiful of conditions, it is abnormal and almost repulsive. We can hardly realise, except through an extensive experience of such cases, that they are quite incapable of effort, and that, moreover, if the brain is clear enough to be aware of its owner's helplessness, that very inability becomes the acutest of trials. Not only to be a burden, but to have knowledge of the fact, that is assuredly the most terrible and humilitating part of the affliction. A thousand minor details, learnt only by those in personal attendance on such sufferers, come into the mind of a qualified nurse; and they certainly ought to suggest themselves with equal significance to parents and guardians. That this is not the case we have sad evidence, for we hear on all sides of young girls being constantly employed in the service of helpless patients. The impropriety of such work being left in such hands hardly seems to need comment; but we may venture to say that the patients themselves ought to be considered first of all in the matter. Surely middle-aged and elderly men and women suffering from all such diseases as we have touched upon, would infinitely prefer the attendance of women of a reasonable age—those who have experience of and sympathy with this helplessness. In Incurable Homes, too, we cannot approve of patients far gone in consumption being constantly associated with young girl-nurses of an age specially susceptible to that dire disease.

The origin of the arrangement which admits such youthful attendants into these special institutions is not far to seek. It is difficult for girls to get nursing work at that age, and therefore after being disappointed at many general hospitals by being told that they cannot be received under twenty-five years old, they turn in despair to such infirmaries or homes as will admit them. In this way probationers are secured at a far lower rate of remuneration than would have to be given to older and seasoned women, who would be far better suited to such onerous duties. We have alluded before to this most important question, and we trust the day will come when the parents and guardians of English girls will fully recognise the dangers and the drawbacks which accompany the employment of such young persons in responsible and exhausting labour amongst the helpless, the insane, and the incurable.

### Everybody's Opinion.

*[Correspondence on all subjects is invited, but we cannot in any way be responsible for the opinions expressed by our correspondents. No communications can be entertained if the name and address of the correspondent is not given, or unless one side of the paper only be written on.]*

### AMERICAN METHODS.

"A Trained and Certificated Nurse" says: I agree with the old proverb "Comparisons are odious," and yet when such invidious ones are made between English and American methods of nursing, I, too, must speak! The art of nursing is growing rapidly amongst our American contemporaries, and without doubt our sisters "over there" are likely enough to overstep us by and by. We have learnt much from them, but they, certainly the best nurses amongst them, frankly own that we have got the start of them in training. When I left England some five or six years go and entered a leading American hospital I seemed to have gone back to the days of my youth. Not that I felt particularly young, only the customs by which I was suddenly surrounded were such antiquated ones. The wards themselves were beautiful, and the bath-rooms, ventilation, &c., appeared perfect, but the nursing arrangements were indeed peculiar. The junior nurse of the ward had, for instance, to scrub out two or three small rooms connected with the wards and to wash soiled linen, &c. On the other hand, the ward-maid or "dining-room maid" took her share in making the patients' beds. One of the nurses left, and was replaced by a fragile-looking girl of twenty who had had no experience, but said herself she "knew all about it, having been a patient in a hospital for six weeks." She took the post of junior nurse, but the scrubbing proved too much and she retired in a week. I had a large experience of the class of women who at that time took up nursing, for fifteen different specimens passed through the ward in a period of three weeks! Truly this was a pleasant experience in a medical ward containing many bad cases including typhoid and pneumonia. It was no unusual thing to catch a nurse (?) telling a typhoid to "sit up and drink off a glass of milk quick." Rigors were never reported to me, save by the patients themselves. Of course, beds made by inexperienced women, including the dining-room maid, were hardly satisfactory. The medical attendance and the quality of the food were alike excellent, but the lack of nurses worthy of the name overbalanced these. I am speaking of 1887, when training was already an established necessity in much-abused old England.

### AMERICAN NURSES.

MRS. G. M. SIBLEY writes: Permit me a few remarks on the subject of American hospital nurses. I read in this week's issue of THE HOSPITAL the report of a somewhat interesting paper, read by Mrs. Bedford Fenwick before the Royal British Nurses' Association on January 20th, entitled "Nursing at the World's Fair." An excellent paper possibly in many respects; surely the authoress's experience of American hospitals must be a limited one, and her visit to the States short. We read a statement to this effect: "In America only well-educated women were admitted into training schools." We all know America is a wonderful world in many respects, and things do grow apace, at a rapidity that is almost bewildering. I am hardly prepared to accept this great change in the hospital world of the States since July, 1891. For two years I have worked with American doctors and visited all the principal hospitals in New York, Washington, Philadelphia, and even cultured Boston for the purpose of attending operations and studying the nursing staff. I can most certainly say I consider the nurses, many of whom I know well, anything but educated women, and a long way below the average educated women we are accustomed to meet in a British hospital. It is an admitted fact by many of the best known American physicians and surgeons, to say nothing of the better educated American people themselves, who have been fortunate enough to have had the experience of British nurses, that is to say, those who are educated and gentlewomen, that these are in every way a superior article to anything they have in the States. Unfortunately, there are in our country many women holding good appointments who could neither lay claim to education or refined manners. We shall next be informed that the American doctor is a superior being to the British one, notwithstanding his two years' curriculum, against the five years of our own medical men. Anyone who has had even a little cosmopolitan experience is quite accustomed to hear the professors in the French and German Universities say a fully qualified American doctor who can afford to spend four or five years working in a European University may learn something of the elements of his profession. Under these existing circumstances, alas, for the educated American nurse who is trained under these men. In conclusion, if in the future we are to hear so much of the new world and its newer hospitals, in justice to the many English gentlewomen nurses I trust the information may be more instructive and more in accordance with the facts.

### PRESENTS AND PRESENTATIONS.

NURSE E. B. writes: I was glad to read your interesting article on "Presents and Presentations." I quite agree with you, as I have often had the same feeling when reading of presentations being made to the matron at a Lying-in Hospital by the nurses. The majority of these are merely pupils for a few weeks. I think the absurdity of giving presents in a case like that speaks for itself. It is clearly to curry favour with a matron who has it in her power, to a certain extent, to assist the nurses in their future career. I venture to make these remarks from personal experience of such an institution.

### PATIENTS' MEALS.

"AN INDIGNANT NURSE" says: I am much pained to hear of a nurse, who has held the position of matron, too, speaking publicly of the patients' food being "hurled at them." I should like to know where such a thing is done? I first worked in a hospital twelve years ago, and then the food of nurses and probationers left much to be desired; but the patients' meals were always served most comfortably. The plates and broth basins were put down to warm quite early, and the patients' diets were given to them hot, and in an appetising manner. I have often heard a young nurse say she wished her food was as satisfactory as that of the patients'. I should certainly be inclined to blame the management of any institution where the meals were given to the patients in any other way.

### "THE HOSPITAL" ENDOWED BED.

NURSE F. L. E. writes: I have received so much encouragement and promised help if the plan, mentioned in THE HOSPITAL of January 14th, is taken up, that I feel sure that other nurses only have to put it before their friends and patients to attain the desired end. You may put my name down for £12, and I hope it will be more, that is if the scheme is taken up.

### Nurses and Cholera.

IF we succeed in keeping cholera away from England the triumph of sanitary science and intelligent organisation will be great. But whether it comes or stays away, it is well that the arrangements with regard to nurses should be complete and sensible ones. All nurses know that they can count on the interest and support of Her Majesty in matters dealing with the common good. The Princess of Wales, too, has given enormous encouragement to nurses by her kindly attitude towards the R.N.P. Fund, and in many other ways. Probably H.R.H. will never know the extent of her influence amongst earnest workers. A comparatively fresh departure is now suggested, and the Lord Mayor has appealed to provincial mayors to support him in securing nurses for the possible advent of cholera. Princess Christian has given her name to the undertaking, which is in itself admirable, so far as it goes. Had there been more practical experience and knowledge on the part of the promoters, and also a little more understanding of institutions, certain initial mistakes would have been avoided! The greatest of these is the entire ignoring of existing hospitals and nursing schools throughout the country. Where are nurses to be obtained except through these? As we pointed out some months ago, women who earn their living at sick nursing cannot be expected to break their engagements and neglect present duties because they may possibly be required by cholera patients by-and-bye. This is what it would amount to if all the best nurses responded to a call to place themselves on one "roll." If women deserted the sufferers who need them now they would assuredly find it a little difficult to get again the work they had thrown up in a moment of enthusiasm.

The Lord Provosts and Mayors throughout the country as business men will recognize the force of what we here urge. Each hospital has its own roll of nurses, and every matron of an institution should be directly communicated with as to exactly how many workers she can provide for any cholera emergency. All trained nurses can be secured through such agencies as these, and the "free-lances," unattached to hospitals, can be invited to co-operate. It is wonderfully unreasonable to ask working women to enrol under other conditions. There are plenty of nurses, doubtless, both for the daily duties and also for the emergency cases, but the most competent ones will never be secured by any association which does not comprehend that the heads of the nursing institutions and general hospitals must be consulted primarily. When the cholera scare, which happily died a natural death last year, was for the time an engrossing topic, hospital authorities were at once on the alert. Not only wards but nurses were promptly "told off," and cholera duty was generally applied for, and was certainly never shirked by anyone worthy to do it. If success is to attend our efforts in the future, every locality must be reached through its existing hospitals and nurses' homes; and co-operation, not competition, should be the watchword enforced by the municipal authorities, who should at once communicate with the hospitals and kindred institutions of their local centres of popul-tion.

### Cholera Precautions.

The Lord Mayor, Alderman Stuart Knill, has addressed the following letter to all the mayors and provosts of the United Kingdom :—"It may be within your worship's knowledge that, when towards the close of August last the country was gravely threatened with an invasion of Asiatic cholera, her Royal Highness the Princess Christian, President of the Royal British Nurses' Association, with that solicitude for the welfare of the suffering for which her Royal Highness is distinguished, issued an appeal to the trained nurses of the United Kingdom to enrol themselves for special services in the nursing of cases of that disease in the event of its establishing itself in the country. At the same time, and through the same initiative, a special committee of the Association was appointed to direct the operations of the nurses thus enrolled and to secure their harmonious co-operation with the various local sanitary authorities under whom they might be called upon to serve.

"At a conference which was held on November 7th last, at the Mansion House, under the presidency of my predecessor in office, and at which her Royal Highness was present, the measures suggested by the special committee received the hearty approval of the Council of the Society of Medical Officers of Health, and among the resolutions which were passed was one to the following effect: 'That the Lord Mayor be invited to take into consideration the expediency and possibility of making the subject of the roll of nurses for cases of cholera known through the agency of the provincial mayors and provosts.'

"As it is the opinion of those best qualified to judge, and among others of the medical officer of the Local Government Board, that the greatest danger may be anticipated to arise in the coming spring and to continue throughout the summer months, and having regard to the supreme importance of making every possible preparation during the intervening interval, I beg to ask your worship to give this matter your consideration, and to invite you, subject to your approval, to take such steps as may seem to you most expedient to bring it under the notice of the sanitary authorities with whom you may be in relation, and of such institutions concerned with the training and employment of nurses as may be within the sphere of your influence.

"I feel persuaded that in so doing your worship would not only aid in furthering her Royal Highness's beneficent design, but also render material service to the country in prospect of a danger the gravity of which it would be difficult to exaggerate."

### Appointments.

Miss Lucy Smith has been made Matron of the Infectious Diseases Hospital, Rochdale. Miss Smith was trained at Pendlebury, and has since held various appointments.

Miss Adeline Griffiths has been appointed Matron at the Metropolitan Asylum District Schools, Darenth. Miss Griffiths held the post of Assistant Matron at the Kensington Infirmary, and afterwards that of Matron at the temporary supplemental infirmary opened by the Guardians in January, 1892. Miss Griffiths' testimonials are excellent.

### Minor Appointments.

Miss Emily Gofton has been appointed Assistant Matron at Kensington Poor Law Infirmary.

### Presentations.

Miss Adeline Griffiths was presented with a handsome marble clock by the officers, nurses, and servants at the Infirmary, Plaistow, on her resignation of the post of Matron at that institution.

### Wants and Workers.

Wanted, a Home for a girl of 15, where she could be medically treated for lupus. Parents can only afford small payment. Reference permitted to *Miss Coleman, 2, Maida Vale, London.*

### For Reading to the Sick.

### THE MESSAGE.

God sends us a message with the New Year, and says, "Trust Me." Perhaps we are longing to make a fresh start, and hardly know how to begin, to us then He says, "Whether your path will be through cloud or sunshine, leave all to Me ; you need not seek to read the future, I know what is good for you, only trust in Me." But we are very sick, and it is hard to take suffering from His hand without a word ; nevertheless, if we lie still our eyes will be surely opened, and we shall clearly see how wise and good He has been to us. In order to get this trust in our Heavenly Father, without which there is no happiness, we must be single-minded. Our dear Lord teaches us that "No man can serve two masters," and St. James says, "A double-minded man is unstable in all his ways." That is the secret of weakness and unrest, of failure and peril ; double-mindedness keeps men back from the task God has set them, and takes the spring and brightness out of their lives, it is the foe to health and growth and peace. It pulls us first one way then another, prompting us to pray that we may bear our trials patiently, but only that we may be on the safe side, making no difference between prayer and selfishness. Our love to God, too, is so half-hearted ; our desire for the blessings of goodness, the joy of the Lord, the gladness of His service, is so inconstant. We may have seen at times that there is no happiness except in serving Him, no happiness except in unselfishness and self-forgetfulness for the sake of others. What restlessness and confusion is brought into our minds by the hopeless toil of living two lives. We spend so much of our strength in vain while we try to work two ways at once. Let us take courage enough to grasp, to hold, and rejoice in God's message, "Trust Me." Yes, trust God in all things. Go patiently on, doing the little works which He has put nearest to our hands. Our dear Lord does not expect great things from us, or He would certainly fit us for doing them ; to be content to lie still in affliction, to keep our motives pure, to have no sordid looking back at those evil inclinations which we have already turned from—these things we cannot fail in if we are single-minded and "trust" God, who will give us that peace which they have who keep His law.

### Notes and Queries.

### Queries.

(24) *Charge Nurses.*—Is it a general rule in hospitals or workhouse infirmaries to move "charge" nurses from one ward to another every three or six months?—*Necropolis.*

(25) *Hospitals Abroad.*—Where can I hear of vacancies in hospitals abroad?—*Nurse Mary.*

(26) *Uniform.*—Will anybody please tell me if aprons are included where uniform is provided?—*Ivy.*

(27) *Rules for Admission.*—Will you tell me the custom in London hospitals regarding admission of nurses already trained?—*Dora.*

(28) *Provincial.*—Can I get an appointment on the staff of a London hospital after provincial training?—*Sister Elizabeth.*

(29) *Bournemouth.*—Can you tell me of any place at Bournemouth where I can go for a week or two at very moderate terms?—*Nurse S.*

(30) *R. N. P. F.*—Where can I get information about this fund?—*Nurse Grace.*

(31) *Colotomy.*—Can you tell me of any Home or Institution which would receive a chronic case of colotomy?—*Nurse Amy.*

(32) *Charles Dickens.*—In which of Dickens' books does he mention the East London Children's Hospital?—*Northumberland.*

### Answers.

(24) *Charge Nurses* (*Necropolis*).—Certainly not—charge or staff nurse is a permanent post. Probationers are moved at regular intervals to secure for them training in the nursing of all kinds of cases. At some institutions staff nurses go on night and day duty alternately, but always in the same ward.

(25) *Hospitals Abroad* (*Nurse Mary*).—Buy the *Hospital Annual*, 1893, 140, Strand, W.C. ; price 5s. Study advertisements in The Hospital. When English nurses are required they are generally advertised for in England.

(26) *Uniform* (*Ivy*).—The amount varies. You can always find out for yourself by writing direct to the matron. See also 25 and 27.

(27) *Rules for Admission.*—Write for "How to Become a Nurse," by Honnor Morten, 140, Strand, price 2s. 6d.; also get particulars from matrons of hospitals and infirmaries.

(28) *Provincial* (Sister Elizabeth).—Yes, it is possible to do so, but most large hospitals now train their own sisters and charge nurses. Probably you could get a post at a good workhouse infirmary.

(30) *R. N. P. F.* (Nurse Grace).—Go and see the Secretary at 8, King Street, Cheapside, and hear all its advantages.

(31) *Colotomy* (*Nurse Amy*).—Please say whether the patient is in a position to pay, and where the Home is required—London or Provincial.

(32) *Charles Dickens* (*Northumberland*).—Do you mean "Our Mutual Friend"?

## Figures and Tables

**Figure f1:**